# Aberrant cervical innate immunity predicts onset of dysbiosis and sexually transmitted infections in women of reproductive age

**DOI:** 10.1371/journal.pone.0224359

**Published:** 2020-01-08

**Authors:** Raina N. Fichorova, Charles S. Morrison, Pai-Lien Chen, Hidemi S. Yamamoto, Yashini Govender, Damilola Junaid, Stanthia Ryan, Cynthia Kwok, Tsungai Chipato, Robert A. Salata, Gustavo F. Doncel

**Affiliations:** 1 Laboratory of Genital Tract Biology, Department of Obstetrics, Gynecology and Reproductive Biology, Brigham and Women’s Hospital, Harvard Medical School, Boston, MA, United States of America; 2 Behavioral, Epidemiologic and Clinical Sciences, FHI, Durham, NC, United States of America; 3 Biostatistics, FHI, Durham, NC, United States of America; 4 University of Zimbabwe, Harare, Zimbabwe; 5 Case Western Reserve University, Cleveland, OH, United States of America; 6 CONRAD, Arlington, VA, United States of America; 7 Eastern Virginia Medical School, Norfolk, VA, United States of America; Massachusetts General Hospital, UNITED STATES

## Abstract

Sexually transmitted infections (STIs) and vaginal dysbiosis (disturbed resident microbiota presenting with abnormal Nugent score or candidiasis) have been associated with mucosal inflammation and risk of HIV-1 infection, cancer and poor reproductive outcomes. To date, the temporal relationships between aberrant cervical innate immunity and the clinical onset of microbial disturbance have not been studied in a large population of reproductive age women. We examined data from a longitudinal cohort of 934 Ugandan and Zimbabwean women contributing 3,274 HIV-negative visits who had complete laboratory, clinical and demographic data. Among those, 207 women later acquired HIV, and 584 women were intermittently diagnosed with *C*. *trachomatis* (CT), *N*. *gonorrhoeae* (NG), genital herpes (HSV-2), *T*. *vaginalis* (TV), candidiasis, and abnormal intermediate (4–6) or high (7–10) Nugent score, i.e. bacterial vaginosis (BV). Immune biomarker concentrations in cervical swabs were analyzed by generalized linear and mixed effect models adjusting for site, age, hormonal contraceptive use (HC), pregnancy, breastfeeding, genital practices, unprotected sex and overlapping infections. High likelihood ratios (1.5–4.9) denoted the values of cervical immune biomarkers to predict onset of abnormal Nugent score and candidiasis at the next visits. When controlling for covariates, higher levels of β-defensin-2 were antecedent to BV, CT and HSV-2, lower anti-inflammatory ratio IL-1RA:IL-1β–to intermediate Nugent scores and candida, lower levels of the serine protease inhibitor SLPI–to candida, lower levels of the adhesion molecule ICAM-1 –to TV, and lower levels of the oxidative stress mitigator and endothelial activation marker VEGF–to NG. Changes in innate immunity following onset of dysbiosis and infections were dependent on HC use when controlling for all other covariates. In conclusion, imminent female genital tract dysbiosis or infection can be predicted by distinct patterns of innate immunity. Future research should characterize biotic and abiotic determinants of this pre-existing innate immunity state.

## Introduction

Sexually transmitted infections (STIs) and vaginal dysbiosis (disturbed resident microbiota) have been associated with risk for HIV-1 infection, cancer and poor reproductive outcomes [1–4]. Evidence points to altered innate immunity and inflammation as major mechanisms underlying these associations [5, 6]. It remains unknown if aberrant host immunity occurs first, predisposing to dysbiosis and STIs and what factors control the temporal relationship between aberrant immunity and imminent infection in reproductive age women.

Among exposures in reproductive age women that have been implicated as immune modifiers at the mucosal portal of HIV, hormonal contraceptives (HC) deserve special attention. HCs are among the most commonly used prescription drugs–used by ~200 million women globally. Of particular concern is accumulating evidence for HIV acquisition risk associated with the use of depot medroxyprogesterone acetate (DMPA), a 3-monthly progestin-only injectable contraceptive and the most commonly used contraceptive method in sub-Saharan Africa [7]–the region that bears an estimated 70% of the global HIV-infection burden [8]. The latest systematic review of epidemiological evidence [9] confirmed that the majority of high-quality observational studies to date support a DMPA association with increased HIV-1 acquisition by comparison to no-HC use. However, a recent randomized controlled clinical trial in Southern and East Africa compared DMPA to another long-lasting progestin (a levonorgestrel implant) and a hormone-free Copper-T intrauterine device and found no significant differences between the three methods in term of HIV risk [10, 11]. However, this trial was powered to detect no less than a 50% increase in HIV risk, did not include a condom only arm or no contraception and did not address potential risks associated with other widely used HC types. Combined oral contraceptives (COCs) that contain both estrogen and progestin are used by ~100 million women worldwide and have also been found to modify genital tract immunity [12, 13]. Although COCs are for now considered safer than DMPA [9], this method was found to increase HIV-1 risk for younger (18–24 years old) participants of the Hormonal Contraceptive and Risk of HIV (HC-HIV) Study–one of the largest prospective investigations of the association between HC use and HIV acquisition to date [14].

Previously, we have shown significant changes in cervical innate immunity in DMPA and COC users participating in the HC-HIV cohort [12]. Among them, we identified immune biomarkers associated with imminent HIV seroconversion including increased levels of RANTES (Regulated on Activation, Normal T cell Expressed and Secreted) and beta-defensin (BD)-2, and decreased levels of soluble leukocyte protease inhibitor (SLPI) [15]. In a previous cross-sectional analysis of HIV-negative visits among cases and controls timed relative to HIV seroconversion, DMPA was associated with RANTES while COC use was associated with proinflammatory mediators with a magnitude and direction of both effects dependent on concomitant abnormal Nugent scores, candidiasis or STIs [13]. The prodromal aspect of these associations remained unknown. To address this gap, we utilized longitudinal specimens from the HC-HIV cohort to test the hypotheses that 1) immune perturbance precedes microbial perturbance, and 2) HC use may contribute to aberrant immunity by modifying the amplitude and direction of cytokine shift at the transition to specific infection or dysbiosis. The HC-HIV longitudinal cohort is especially suitable for these analyses since it collected at each study visit comprehensive information on the covariates known to affect cervical immunity such as age, pregnancy, breastfeeding, HC use, vaginal practices and unprotected sex.

## Materials and methods

### Human subject protection

The study was carried in accordance with the Code of Ethics of the World Medical Association (Declaration of Helsinki). The parent HC-HIV study was carried with subjects’ informed consent and Institutional Review Board approval for human subject research at participating institutions in the United States and Africa. The biomarker sub study protocol received a nonhuman subject determination (use of deidentified data) from the Office of International Research Ethics at FHI 360 and the Institutional Review Board at Brigham and Women's Hospital.

### Population

This study utilized cervical specimens collected from 18–35 years old participants (n = 934) enrolled in the HC-HIV study from family planning clinics in Uganda (n = 293) and Zimbabwe (n = 641) [16]. The HC-HIV study was originally designed to investigate the risk of HIV in users of DMPA and COC compared to women not using HC including using no contraceptives at all [17, 18]. The analyses presented here encompassed only the HIV-negative visits with 3274 available cervical swabs collected quarterly [12]. The demographic characteristics of the 934 participants including age, pregnancy, breastfeeding, number of sexual partners, use of condoms and vaginal practices, are presented in supplemental S1 Table assessing all variables at two partially overlapping baselines–one timed to incidence of dysbiosis and STIs and the other timed to two quarterly visits prior to HIV seroconversion. Most (96.5%) of the specimens were obtained during a 2-year follow-up. Based on their own preference, women chose to use—either DMPA (150 mg injected every 3 months) or COCs (30 mcg ethinyl estradiol and 150 mcg levonorgestrel), or no hormonal contraception. For the purposes of statistical analyses, DMPA or COCs designation was based on the method used for the majority of time between their previous study visit and the selected visit. Study staff administered DMPA injections and dispensed COCs pills. No specimens were collected during menstrual bleeding and friable cervix/visible blood was rarely recorded during swab collection. Sociodemographic information was collected at the baseline visit. Behavioral surveys were completed at each visit covering the 3 months prior to the study visit. STIs/HIV diagnostic was performed at each visit.

### Laboratory methods assessing infection and immunity

*C*. *trachomatis* (CT) and *N*. *gonorrhoeae* (NG) were diagnosed by PCR (Roche AMPLICOR), herpes simplex virus type 2 (HSV)-2 –by antibody ELISA, and *T*. *vaginalis* (TV) and *Candida–*by wet mount. Abnormal microbiota and bacterial vaginosis (BV) were assessed by Nugent scoring. HIV status was determined by ELISA and confirmed by PCR. The rate of co-infections is shown in supplemental S2 Table. Co-infections appeared randomly distributed among each dysbiotic condition and STI except a higher rate of CT-NG infections than co-infections of CT or NG with other pathogens and rarer BV-candida co-occurrence compared to co-occurrence of each of this dysbiotic conditions with STIs (S1 File).

Soluble immune mediators were quantified in elutions from cervical swabs collected in Amplicor buffer (Roche Diagnostics). The Amplicor elutions were shipped frozen to the Laboratory of Genital Tract Biology, Brigham and Women’s Hospital, where they were further processed and analyzed for protein levels of the following 10 immune biomarkers as previously described [12, 13, 19]: Interleukin (IL)-1β, IL-6, IL-8 (CXCL-8), IL-1 receptor antagonist (IL-1RA), RANTES (CCL-5), macrophage inflammatory protein (MIP)-3α (CCL-20), vascular endothelial factor (VEGF), and soluble intercellular adhesion molecule (ICAM)-1 (CD54), soluble leukocyte protease inhibitor (SLPI) and BD-2. These biomarkers were chosen for their established role in vaginal inflammation and HIV risk and reliable detection in cervical secretions, including cervical Amplicor elutions [13, 19–21]. Reproducibility was confirmed by running a quality control split sample on each assay plate, which showed <25% inter-assay coefficient of variation (for individual markers as follows: SLPI—8.6%, BD-2–13.8%, IL-1RA—19.3%, ICAM-1 20.2%, IL-1β - 5.3%, IL-6–8.8%, IL-8-4.9%, MIP-3α - 9.9%, RANTES 6.5%, and VEGF 14.2%).

### Statistical analysis

#### Definitions of cervicovaginal infection (CVI) status

Women who remained CVI negative (normal Nugent score, no candida and no STIs) throughout the study and contributed cervical samples on at least two consecutive non-missing quarterly visits were assigned for the purposes of this analyses to the category of the “CVI-free” control. Incident infection was defined as any new CVI diagnosed during the study in a woman who had at least two consecutive non-missing quarterly visits tested with a positive visit preceded by a negative visit for that specific incident CVI.

#### Statistical methods

All statistical analyses were performed using SAS 9.4 (SAS Institute, Cary, NC); p-values <0.05 were considered statistically significant.

Because levels of cervical immune mediators did not follow Gaussian distribution, concentrations were normalized using Box-Cox power transformation prior to analyses. We used generalized mixed effect models to estimate mean levels of individual biomarkers and differences between visits by HC and CVI status, as well as individual level changes from pre- to post-infection visits at the time of an incident CVI. We used generalized linear models with the generalized estimating equation approach to estimate odds ratios (OR) and 95% confidence intervals of top and bottom quartile concentrations of immune mediators at CVI-negative visits preceding an incident CVI and at the CVI positive visits. We used unstructured covariance structure for both generalized linear models and generalized mixed effect models for data analysis because no assumptions were needed. Top or bottom quartile were chosen because they are commonly used to examine the effect of the opposite extremes in deviation from the median, levels at which immune imbalance is most likely to have biological significance. Baseline demographic characteristics were compared by the Cochran-Mantel-Haenszel test; Fisher’s exact test was used when a comparison group comprised ≤ 5 women. Binomial/multinomial tests were used for tests of proportions based on an asymptotic Wald test to calculate the p-values. The Z test was applied to evaluate the statistical significance of likelihood ratios (LR) to assess the value of top quartile biomarker concentrations for predicting each CVI outcome at the next quarterly visit using the contingency table approach with an asymptotic Wald test to calculate the p-values.[22]. LR were also calculated to estimate the value of each CVI for predicting another incident CVI at the next quarterly visit.

#### Adjustment for covariates

Although pregnancy was an exclusion criterion at enrollment, a significant number of women (127, 13.6%) became pregnant within the 2-year period of the study, fewer among HC users than non-HC users. Because pregnancy itself has been associated with changes in cervical immunity, we controlled for pregnancy in our covariate adjustment in the generalized mixed effect and generalized liner models. Other covariates controlled for in these analyses were country, age, breastfeeding, overlapping CVIs, HC use, unprotected sex and vaginal practices as they have all been associated with variations in cervicovaginal cytokine levels [12, 23]. For comparison of HC methods, visits were stratified by HC use and controlled for the remaining variables.

## Results

### Aberrant cervical immunity precedes incident vaginal dysbiosis and STIs

To test the hypothesis that pre-existing aberrant cervical immunity (may predispose to incident infection or dysbiosis, we calculated the value of top quartile levels of clinically validated innate immune biomarkers to predict a new infection or dysbiotic condition using likelihood ratios (LR) (Table 1). Top quartile levels of all 10 individual immune mediators (except the anti-inflammatory ratio IL-1RA:IL-1β) predicted Nugent scores of 4–6 as well as BV at the next visit with higher than random occurrence (LR 1.8–4.88, p<0.05). Top quartile levels of eight of those individual mediators predicted candida occurrence at the next visit (LR 1.5–2., p<0.05). Top quartile levels of the same 10 individual immune mediators tended to convey a negative predictive value (LR <1) for all STIs, which reached significance for select markers predicting incident CT (IL-6) and HSV-2 (IL-6, SLPI and ICAM-1 (LR = 0.10–0.04, p<005). Top quartile levels of the IL-1RA:IL-1β ratio conveyed less than random occurrence of CVIs, which reached significance for incident HSV-2 (LR = 0.11, p<0.05).

**Table 1:** Likelihood ratios (LR) estimating the value of top quartile biomarker concentrations to predict incident cervicovaginal infection (CVI) outcomes at the next quarterly visit. A value of 1 indicates a random occurrence of an incident CVI, e.g. vaginal dysbiosis or sexually transmitted infection. Values ≠ 1 indicate non-random distribution and are highlighted if >1 (higher likelihood, orange) or <1 (lower likelihood, blue).

	Predicted Incident CVI Outcomes						
	Vaginal dysbiosis			Sexually Transmitted Infections			
Predictive Biomarker: Top quartile levels at the visit prior to CVI incidence	Nugent 4–6 (n = 445)	BV (n = 477)	Candida (n = 271)	NG (n = 71)	TV (n = 82)	CT (n = 58)	HSV-2 (n = 36
**IL-1β**	**4.67**^**a**^	**3.22**	**2.39**	0.44	0.44	0.28	0.22
**BD-2**	**3.74**	**4.21**	**2.42**	0.37	0.58	0.53	0.37
**RANTES**	**2.95**	**3.09**	**2.45**	0.64	0.27	0.36	0.27
**MIP-3α**	**4.88**	**3.35**	**2.35**	0.53	0.29	0.24	0.24
**IL-6**	**3.91**	**2.91**	**1.74**	0.39	0.30	***0*.*04***^***b***^	***0*.*09***
**IL-8**	**3.77**	**2.77**	**1.95**	0.41	0.36	0.23	0.27
**SLPI**	**3.00**	**2.03**	**1.55**	0.34	0.28	0.14	***0*.*10***
**IL-1RA**	**2.67**	**1.87**	**1.50**	0.30	0.33	0.23	0.13
**ICAM-1**	**2.53**	**1.80**	1.40	0.50	0.23	0.33	***0*.*10***
**VEGF**	**3.18**	**2.00**	1.21	0.25	0.29	0.25	0.25
**IL-1RA:IL-1β**	0.89	1.02	0.60	0.21	0.28	0.16	***0*.*11***

^a^Values of significantly higher chance/likelihood of incident CVI at the next visit (≥1.5 based on 2-sided z score test with a 0.05 significance level) are highlighted with darker shade and bolded.

^b^Values of significantly lower chance/likelihood of incident CVI at the next visit (≤0.11, p<0.05, 2-sided z -score) are highlighted in darker shade, bolded and italicized.

Since the Z test does not control for co-variates, we wanted to determine if any other infection preceding each incident CVI might explain the predictive value of aberrant immunity (Table 2). The predictive value of such CVIs was limited to incident Nugent 4–6 (predicted by BV, LR 3.7), BV (predicted by Nugent 4–6, LR 5.08, and candida, LR 1.7), NG (predicted by CT, LR 3.34), and HSV-2 (predicted by BV, LR 2.54). None of the CVIs predicted incident CT and candida in our sample as did immune biomarkers, suggesting that the predictive value of innate immunity is not likely a simple proxy for preceding clinically diagnosed CVIs.

**Table 2:** Likelihood ratios (LR) estimating the value of a specific cervicovaginal infection (CVI) to predict a new incident CVI^a^ at the next visit based on 2-sided z-score test with p<0.05 significance level.

	Predicted Incident CVI Outcome						
	Incident Vaginal Dysbiosis			Incident Sexually Transmitted Infection			
Predictive Marker: CVI at the visit preceding the incidence of the CVI listed on the right	Nugent 4–6 (n = 450)	BV (n = 484)	*Candida (n = 278)*	*NG (n = 72)*	*TV (n = 82)*	*CT (n = 58)*	HSV-2 (n = 37)
Nugent 4–6	** **	5.08**	1.01	0.73	1.61	0.55	0.94
Nugent 7–10 (BV)	3.70**	** **	1.23	1.14	0.97	1.67	2.54*
*Candida*	1.07	1.70*	** **	1.85	1.13	0.91^b^	1.75^b^
*N*. *gonorrhoeae (NG)*	0.85	0.86	1.43	** **	0.88^b^	2.01^b^	2.o8^b^
*T*. *vaginalis (TV)*	1.20	1.27	1.28	0^b^	** **	0.97^b^	0^b^
*C*. *trachomatis (CT)*	0.90	0.80	0.71^b^	3.34*^b^	1.3^b^	** **	3.07^b^
HSV-2	1.08	1.00	1.20	1.07	0.98	1.18	** **

LR = 1 indicates a random occurrence of a CVI incident e.g. vaginal dysbiosis or sexually transmitted infection. LRs > 1 indicate higher and <1 lower likelihood of incident CVI.

*p<0.05

**p<0.01.

^a^Incident CVI visit is defined as a CVI-positive visit diagnosed following a non-missing quarterly visit, which was negative for the same CVI.

^b^The Fisher exact test was used instead of the z-score test due to the small number of cases (<5) in the category examined.

**Fig 1:**
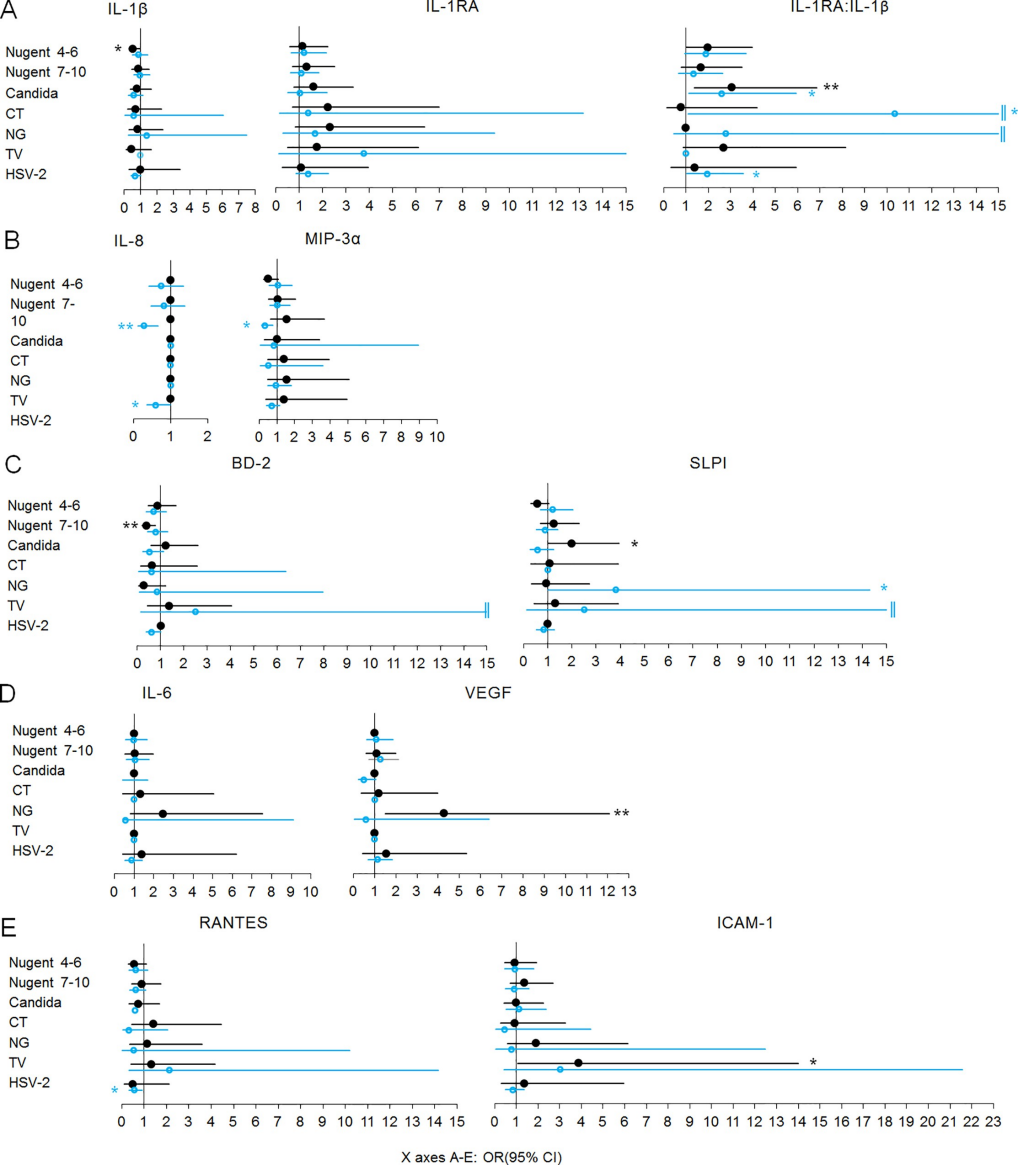
Odds ratios (OR) of having bottom quartile concentrations of mediators of inflammation based on imminent or current infection controlling for site, age, hormonal contraceptives, pregnancy, breastfeeding, overlapping infections, unprotected sex and vaginal practices: A) regulators of IL-1 receptor signaling; B) proinflammatory chemokines; C) anti-microbial effectors; D) mediators mitigating oxidative stress; E) mediators of lymphocyte traffic. OR and 95% confidence intervals (CI) (X axis) for having bottom quartile levels of each biomarker compared to all visits by women who remained negative for the cervicovaginal infections (n = 134) listed in the Y axis: black color, filled circles, marks OR (95% CI) and p value symbols for the quarterly visit preceding CVI; blue color, open circles, marks OR (95% CI) and p value symbols for any visit positive for CVI; *, p<0.05, **, p<0.01; ‖, upper CI>15. Lack of CI indicates not enough visits with bottom quartile distribution with each characteristic tested. CT = *C*. *trachomatis*, NG = *N*. *gonorrhoeae*, TV = *T*. *vaginalis*. Visits with any missing diagnostic test were excluded. Number of incident infections: Nugent 4–6 = 252, BV = 273, candida = 160, NG = 39, TV = 36, CT = 32, and HSV-2 = 19. Number of prevalent infections: Nugent 4–6 = 651, BV = 989, candida = 232, NG = 95, TV = 96, CT = 83, and HSV-2 = 1778.

**Fig 2:**
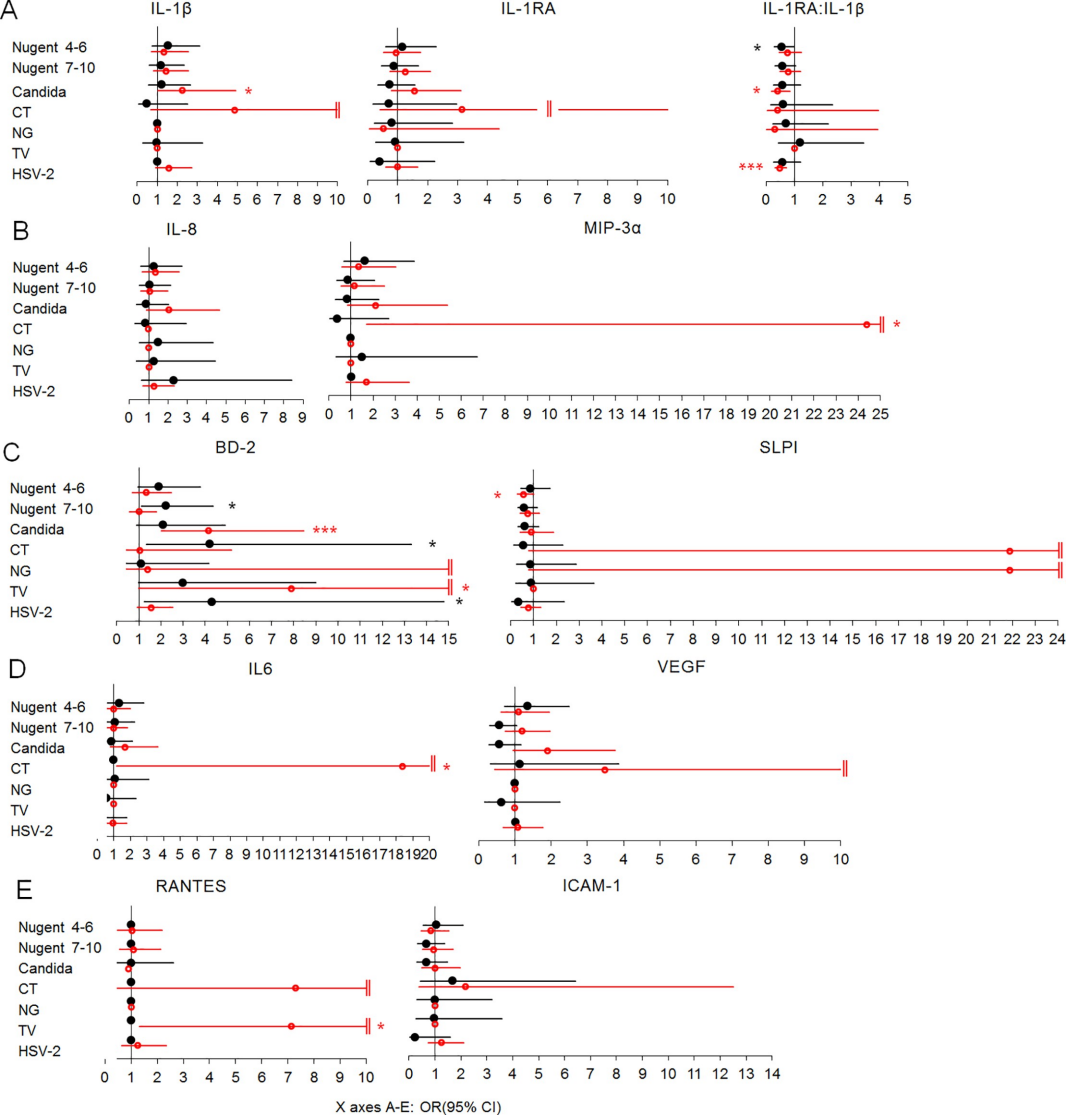
Odds ratios (OR) of having top quartile concentrations of mediators of inflammation based on imminent or current infection controlling for site, age, hormonal contraceptives, pregnancy, breastfeeding, overlapping infections, unprotected sex and vaginal practices: A) regulators of IL-1 receptor signaling; B) proinflammatory chemokines; C) anti-microbial effectors; D) mediators regulated by oxidative stress; E) mediators of lymphocyte traffic. OR and 95% confidence intervals (CI) (X axis) for having top quartile levels of each biomarker compared to all visits by women who remained negative for the cervicovaginal infections (n = 134) listed in the Y axis: black color, filled circles, marks OR (95% CI) and p value symbols for the quarterly visit preceding CVI; red color, open circles, marks OR (95% CI) and p value symbols for any visit positive for CVI; *, p<0.05, **, p<0.01, ***, p<0.001; ‖, upper CI>15. Lack of CI indicates not enough visits with bottom quartile distribution with each characteristic tested. CT = *C*. *trachomatis*, NG = *N*. *gonorrhoeae*, TV = *T*. *vaginalis*. Visits with any missing diagnostic test were excluded. Number of incident infections: Nugent 4–6 = 252, BV = 273, candida = 160, NG = 39, TV = 36, CT = 32, and HSV-2 = 19. Number of prevalent infections: Nugent 4–6 = 651, BV = 989, candida = 232, NG = 95, TV = 96, CT = 83, and HSV-2 = 1778.

To further separate the effect of aberrant immunity from that of other covariates, we examined the probability of having bottom quartile (Fig 1) and top quartile (Fig 2) biomarker concentrations at the CVI-negative quarterly visits preceding an incident CVI as well at all CVI-positive visits when controlling for site, age, HC use, pregnancy, breastfeeding, overlapping CVIs, unprotected sex and vaginal practices. The comparator for both the prodromal CVI-negative visits and the prevalent CVI visits were all the visits contributed by the CVI-free women (n = 134).

Distinct patterns of significantly aberrant immunity (top and/or bottom OR, 95% CI, p<0.05) preceded each individual incident CVI as follows (denoted by black symbols in Figs 1 and 2): intermediate Nugent score was preceded by higher IL-1β (bottom quartile OR 0.52, 0.28–0.97) and lower IL-1RA:IL-1β (top quartile OR 0.54, 0.29–0.98); BV was preceded by higher BD-2 (top quartile OR 2.22, 1.13–4.37 and bottom quartile OR 0.39, 0.20–0.78); candida was preceded by lower IL-1RA:IL-1β (bottom quartile OR 3.06, 1.37–6.84) and lower SLPI (bottom quartile OR 2.01, 1.02–3.94); CT and HSV-2 were preceded by higher BD-2 (top quartile OR 4.22, 1.34–13.32 and 4.32, 1.26–14.79, respectively), TV was preceded by lower ICAM-1 (bottom quartile OR 3.89, 1.08–14), and NG was preceded by lower VEGF (bottom quartile OR 4.28, 1.52–12.05).

Overall, prevalent CVIs (denoted by blue symbols in Fig 1 and red symbols in Fig 2) could also be distinguished by the host response to infection, several of them contributing to patterns previously identified as prodromal to HIV infection in this population of women [15] e.g. higher RANTES (TV, top quartile OR 7.14, 1.31–39.03), and HSV-2 (bottom quartile OR 0.56, 034–0.92), higher BD-2 (TV, top quartile OR 7.89, 1.01–61.64, and candida, top quartile OR 4.14, 2.03–8.46) and lower SLPI (intermediate Nugent score, top quartile 0.55, 0.3–1), and NG, (bottom quartile OR 3.81, 1.02–14.29). Some trends of aberrant immunity such as increase in BD-2, prodromal to TV infection (top quartile 2.99, 1–9), and reduced IL-1RA:IL-1β (bottom quartile OR 3.06, 1.37–6.84), prodromal to candida, persisted or exacerbated after the onset of these CVIs (top quartile OR for BD-2 7.89, 1.01, 61.64 in prevalent TV, and top quartile OR for IL-1RA:IL-1β 0.40, 0.19–0.84 in prevalent candida) suggesting that innate immunity state preceding infection may contribute to inflammation and risks associated with these prevalent CVIs.

### Immune responses to incident dysbiosis and STIs are modified by HC use

Our previous cross-sectional analysis of 894 samples timed to HIV seroconversion identified association between prevalent CVIs and levels of the biomarkers, identified here as predictors of CVIs, and this association was dependent for its direction and amplitude on HC use (DMPA, COC versus no-HC) [13]. We now confirmed these observations with the increased longitudinal size of 3,274 samples (supplemental S3 Table). However, the cross-sectional nature of the prior analyses precluded investigation on whether the HC effects were due to baseline immune differences among the women with imminent CVIs or due to differential impact on responses to CVI pathogens.

**Fig 3:**
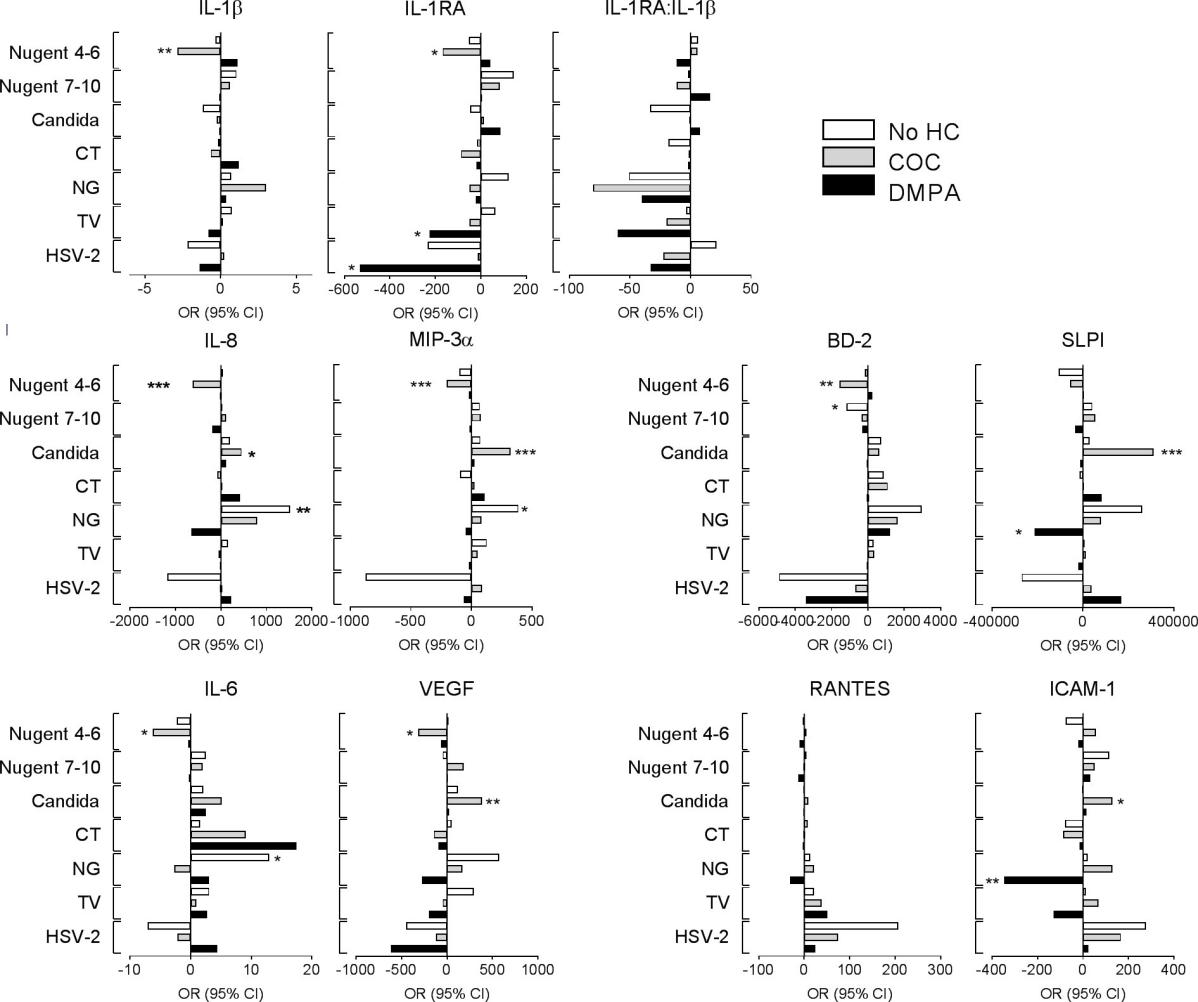
Changes in cervical immunity from before to after acquisition of cervicovaginal infections (CVIs) stratified by hormonal contraceptive use (no HC, DMPA and COC) and adjusted for site, age, pregnancy, breastfeeding, overlapping CVIs, unprotected sex and vaginal practices. CVIs include: (1) vaginal dysbiosis defined by Nugent 4–6, Nugent 7–10 or a positive test for candida, and (2) sexually transmitted infections positive for *Chlamydia trachomatis* (CT), *Neisseria gonorrhoeae* (NG), *Trichomonas vaginalis* (TV) or genital herpes (HSV-2). Horizontal axis represents averages of individual level differences in pg biomarker per ml cervical elution adjusted to mg/ml total protein. The concentration of each biomarker at the visit preceding each CVI is set to 0 on the X axis of each bar plot; bars to the left represent decreased and bars to the right–increased levels at the time of CVI incidence. Number of incident infections: Nugent 7–10 –no HC = 56, DMPA = 98, COC = 97, Nugent 4–6 –no HC = 53, DMPA = 107, COC = 112, TV–no HC = 12, DMPA = 13, COC = 11, Candida—no HC = 33, DMPA = 66, COC = 68, CT–no HC = 9, DMPA = 10, COC = 13, GC–no HC = 11, DMPA = 18, COC = 10, HSV-2 –no HC = 5, DMPA = 5, COC = 8. Visits with any missing diagnostic test were excluded. *, p<0.05, **, p<0.01, ***, p<0.001.

Controlling for HC in the longitudinal analysis (Figs 1 and 2) did not eliminate differences in immune profiles preceding CVIs suggesting that at least in part HC may affect responses to dysbiosis and STI pathogens. We hypothesized that HC use may contribute to aberrant immunity by modifying the amplitude and direction of cytokine shift at the transition to specific infection or dysbiosis. To address this hypothesis, we assessed the change in immune mediator levels from the visit preceding each CVI to the incident CVI visit, stratifying by HC use and controlling for site, age, pregnancy, breastfeeding, overlapping CVI, unprotected sex and vaginal practices. Again, distinct patterns of significant changes emerged which varied by HC use (Fig 3).

The most significant changes in biomarker levels were observed among COC users. This happened when COC users were transitioning from normal to abnormal Nugent score 4–6, which was associated with a broadly immunosuppressive response (decreased IL-1β, IL-1RA, IL-8, MIP-3α, IL-6, VEGF and BD-2), and also when they acquired candidiasis, which was associated with a broadly proinflammatory response (increased IL-8, MIP-3α, SLPI, VEGF and ICAM-1). In contrast, immunosuppressive responses were associated with DMPA use in NG infection (decreased SLPI and sICAM-1). Immune responses that may contribute to clearance of bacterial infection in general (increased IL-6, IL-8 and MIP-3α) were detected when acquiring NG but were only statistically significant in no-HC users.

## Discussion

This study is the first to establish patterns of pre-infection cervical immunity that are predictive of vaginal dysbiosis (abnormal intermediate Nugent score, BV and candidiasis) and specific STIs, and to describe the effect of hormonal contraceptives on shaping host immune responses to acquiring these pathogenic conditions.

### Innate immunity precursory to incident CVI

A novel mechanistic finding of our study was that CVI outcomes are consequent to an aberrant (significantly deviant from the median) cervical immunity detectable within 3 months prior to CVI status change. Aberrant immunity preceded and predicted incident CVIs.

Top quartile levels of all 10 individual immune mediators measured in cervical specimens predicted incidence of both milder (intermediate Nugent score 4–6) and more advanced (BV-associated Nugent scores 7–10 and candidiasis) vaginal dysbiosis using LR zeta score test. Positive predictive values (LR>1) of top quartile biomarker levels for each of these outcomes at the next visit we confirmed with significant LRs ratios ranging from 1.5 to 4.88. In contrast, suppressed cervical immunity (top quartile levels with LR<1) appeared predictive of imminent HSV-2 seroconversion, especially significant for the antimicrobial effector SLPI and interferon-regulated anti-viral immune response mediators IL-6 and sICAM-1 (top quartile likelihood ratios 0.09–0.10). Lower IL-6 was also predictive of a subsequent CT infection (top quartile likelihood ratio 0.04). Immune biomarkers but not any other CVI were uniquely predictive of candida and CT.

Odds ratios for top quartile levels of several immune mediators remained significantly associated with imminent CVIs after controlling for HC and other potential confounders including country, age, overlapping CVIs, pregnancy, breastfeeding, unprotected sex and vaginal practices, suggesting that other factors, not adjusted for in our study, may be at play preconditioning baseline immunity. Metagenomic analyses of the cervicovaginal microbiota has begun to identify taxonomic units and potential new pathogens associated with cervicovaginal immunity [1, 3, 24]. It is possible that subtle or subclinical vaginal microbiome variations—some of them attributable to HC use [25]—precondition pre-infection immunity and thus could impact susceptibility to infection.

In this study, each CVI was associated with a distinct prodromal state of cervical innate immunity, with higher BD-2 shared by imminent BV, CT, and herpes, reduced IL-1RA:ILβ shared by imminent intermediate Nugent score and candida, while higher IL-1β and lower VEGF, SLPI, and ICAM-1 being uniquely precursory for intermediate Nugent score, NG, candida, and TV, perhaps reflecting distinct pathogenic traits of vegetative bacterial, invasive bacterial, fungal and extracellular parasitic infections, respectively.

Among the 11 biomarkers measured in the HC-HIV study, BD-2 emerged as a top predictor of CVI outcome, higher both prior to three CVIs and after onset of prevalent candida and TV. BD-2 is a cationic peptide with antimicrobial activity [26] and chemotactic properties [27], in particular for dendritic cells [26], which may constitute a mechanism for facilitating HIV infection. In our cross-sectional analysis we had identified higher cervical BD-2 levels in women who became HIV positive at the next visit compared to those who remained HIV negative throughout the study [12] and was one of few single biomarker predictors of HIV seroconversion in our longitudinal analysis [15]. It was predictive of dysbiosis by the LR test and after controlling for all covariates, including HC, top quartile levels of BD-2 remained significantly associated with 3/7 CVIs tested. BD-2 is part of the protective host responses to infection but it is possible that its prodromal upregulation is simply an epiphenomenon or reporter of an underlying undiagnosed microbiome shift or asymptomatic STI (e.g. HPV, CMV or mycoplasma) at the visit preceding incident BV, CT and herpes. BD-2 upregulation has been recently associated with HPV16 [28]. With prevalent CVI, BD-2 stood up also with high OR for top quartile levels significant for TV and candida and trending for other CVIs. Further research on BD-2 is needed to understand the molecular mechanism underlying its predictive value for CVIs and potential microbiome changes.

Having higher levels of RANTES has also emerged as a significant predictor of HIV acquisition risk. In our sample, RANTES was predictive of dysbiotic state but not after adjustment for confounders. It was also higher in prevalent TV and herpes consistent with their association with increased HIV risk. Higher RANTES was associated with DMPA in a manner dependent on prevalent CVI and increased in prevalent CT and HSV-2 only in no-HC users (S3 Table). Although the primary function of RANTES as a chemokine is to recruit immune cells to sites of infection or injury [29], it has pleiotropic roles and, depending on a concentration threshold, it may engage different receptors with either protective or pro-HIV activation outcomes [30]. Thus, maintaining RANTES at lower physiologic ranges may be especially critical.

Lower SLPI, another marker of HIV risk, was prodromal for candida and emerged significantly in DMPA users at the time of NG incidence (Fig 3) and in overall prevalent TV and NG (S3 Table). On the other hand, SLPI was increased in COC users both at the time of CVI incidence and in prevalent STIs (S3 Table), thus perhaps counterbalancing the overall proinflammatory effect of COC due to increase in other proinflammatory mediators in the context of intermediate Nugent, BV, candida, TV and HSV-2 (Fig 3 and S3 Table). SLPI is an antimicrobial effector and anti-inflammatory mediator upregulated by inflammation with anti-fungal properties which may explain its impact on candida [31]. The individual roles of IL-6, VEGF and ICAM-1, which are markers of oxidative stress, endothelial activation, and anti-viral stress response [32], in preconditioning imminent CVI is also intriguing and should be further explored in experimental models of these CVIs and as well as in the context of microbiome changes and especially the understudied vaginal virome.

### Effect of HC use on host immune responses to incident CVIs

With high statistical power (based on 3,274 visits contributed by 934 women) we established longitudinal pre- and post-infection differences in levels of cervical biomarkers associated with specific CVI outcomes and confirmed prior cross-sectional findings of dependence of these patterns on use of DMPA and COC [13]. The HC-CVI interaction was evident from the differences in proinflammatory, anti-inflammatory and microbicidal cervical biomarkers, when women were stratified by both HC use and CVI status and compared to women who were CVI-free and did not use HC. Across all visits, DMPA and COCs were associated with altered cervical immunity dependent on CVI examined. These findings begged the question of whether immunologic changes preceded/predisposed women to infection or were the results of altered responses to pathogens after CVI was established and diagnosed.

A new mechanistic finding of the current analysis was that HC use may influence protective host responses to infection at the time of CVI incidence, which may delay or disable clearance of bacterial as well as viral infections. Immune responses varied by HC use for incident CVI. In COC users they were particularly proinflammatory (5/10 biomarkers), when transitioning to candidiasis, and immunosuppressive (7/10 biomarkers), when transitioning to dysbiosis indicated by Nugent score. In DMPA users, responses were immunosuppressive, particularly significant in diminishing markers of effector function for incident gonorrhea (decrease of the antimicrobial protein SLPI and the regulator of lymphocyte trafficking ICAM-1), and anti-inflammatory function at HSV-2 seroconversion (decrease in IL-1RA). These findings warrant further prospective studies to establish, in depth, the role of HC use and hormonal levels in delaying, disabling, or altering clearance of CVIs. DMPA and COCs had opposing effects on the transition of cervical immunity from a CVI negative to a CVI-positive status which is in line with differences observed by cross-sectional [12] and prevalent (S3 Table) analyses. These differences between HC types might be expected, based on different steroid receptor utilization and downstream differential regulation of inflammatory pathways by the two different progestins–DMPA and levonorgestrel in COC, which have been well established in vitro and in pharmacokinetic studies [33–39]. How the different utilization of steroid receptors may impact pathogen-specific host immune responses at the molecular level remains to be determined. In addition, DMPA is a progestin-only HC while COCs are progestins combined with estrogens, therefore differences in the HC formulations could also account for the different immunological responses, including differential regulation of BD-2 [40]. What is intriguing and still requires elucidation is that both DMPA and COCs may act in the same direction immunologically but only in the presence of certain microbes. This phenomenon requires further investigation.

### Limitations and strengths of the study

Among the strengths of our study are the large sample size, longitudinal design and well established and validated technologies for biomarker assessment by a central laboratory accredited by the College of American Pathologists. The original HC-HIV study was powered to achieve 87% power at the significance level of 0.05 to detect an increase in the probability of becoming HIV-1 infected when an immune mediator is increased by one standard deviation in ~200 HIV infected cases and HIV negative controls. We did not target a prespecified clinical effect size for the hypothesis that cervical immunity will predict acquisition of other STIs or vaginal dysbiosis. However, we had enough power to detect statistically significant changes in immune biomarkers from before to after abnormal Nugent score (496 women, 533 conversions) and candidiasis (161 women, 172 incidences) for which activation of any of the immune biomarkers was predictive of the microbiologic change.

The possibility of misreporting of HC use dictates caution when interpreting effects of HC in population studies [41]. Notably, in the HC-HIV study, the source of our study samples, the clinical research staff administered all DMPA injections and provided the oral hormonal contraceptive pills to the subjects at each study visit, which may have reduced issues with compliance and misreporting, which cannot be completely overcome even in randomized controlled clinical trials. Furthermore, soon to be published, most recent data from the HC-HIV study presented at the HIVR4P conference confirmed effective suppressing of endogenous β-estradiol and progesterone in both DMPA and COC users, suggesting effective compliance with the HC majority method as assigned for the analyses presented here.

The biomarkers that we examined in this study were ten proteins with largely non-redundant functions carefully pre-selected prior to any laboratory work being done based on immunologic knowledge and principles. Because this is a very different situation from an exploratory study that randomly selects a sample from a very large group (i.e., thousands) of markers or gene analysis without clearly targeting them *a priori*, adjustment of p values for multiple comparisons would unnecessarily increase type II error and was omitted in accordance with well-accepted statistical analysis criteria [42]. To minimize type I error, we examined each of these proteins independently. The predictive value of each individual protein should be validated in future diverse cohorts.

A limitation of our study is that, due to the high baseline prevalence of STIs among our research subjects, a relatively smaller number of women acquired new infections by HSV-2, *C*. *trachomatis*, *T*. *vaginalis* and *N*. *gonorrhea* during the study. This limited statistical power to predict incident infections by immune activation and hence increased the possibility of Type 2 error. Therefore, negative findings about incident herpes, chlamydiasis, gonorrhea and trichomoniasis should be interpreted with caution. Furthermore, the laboratory diagnosis of trichomoniasis and yeast infections relied on in-clinic wet mount, which allows immediate treatment and has high specificity, but lower sensitivity compared to newer molecular diagnostic tests [43–45]. Therefore, we cannot exclude the possibility that undiagnosed subclinical infections by these pathogens might have contributed to the prodromal state of cervical immunity. A further limitation is that biomarkers of semen exposure were not available for the HC-HIV study. We relied on self-reported condom use which is traditionally flawed by misreporting. However, none of the current biomarkers including Y chromosome DNA and semen antigens provide ideal proof for unprotected sex due to limited sensitivity relative to timing of sampling within 24h-exposures.

### Conceptual paradigm

**Fig 4:**
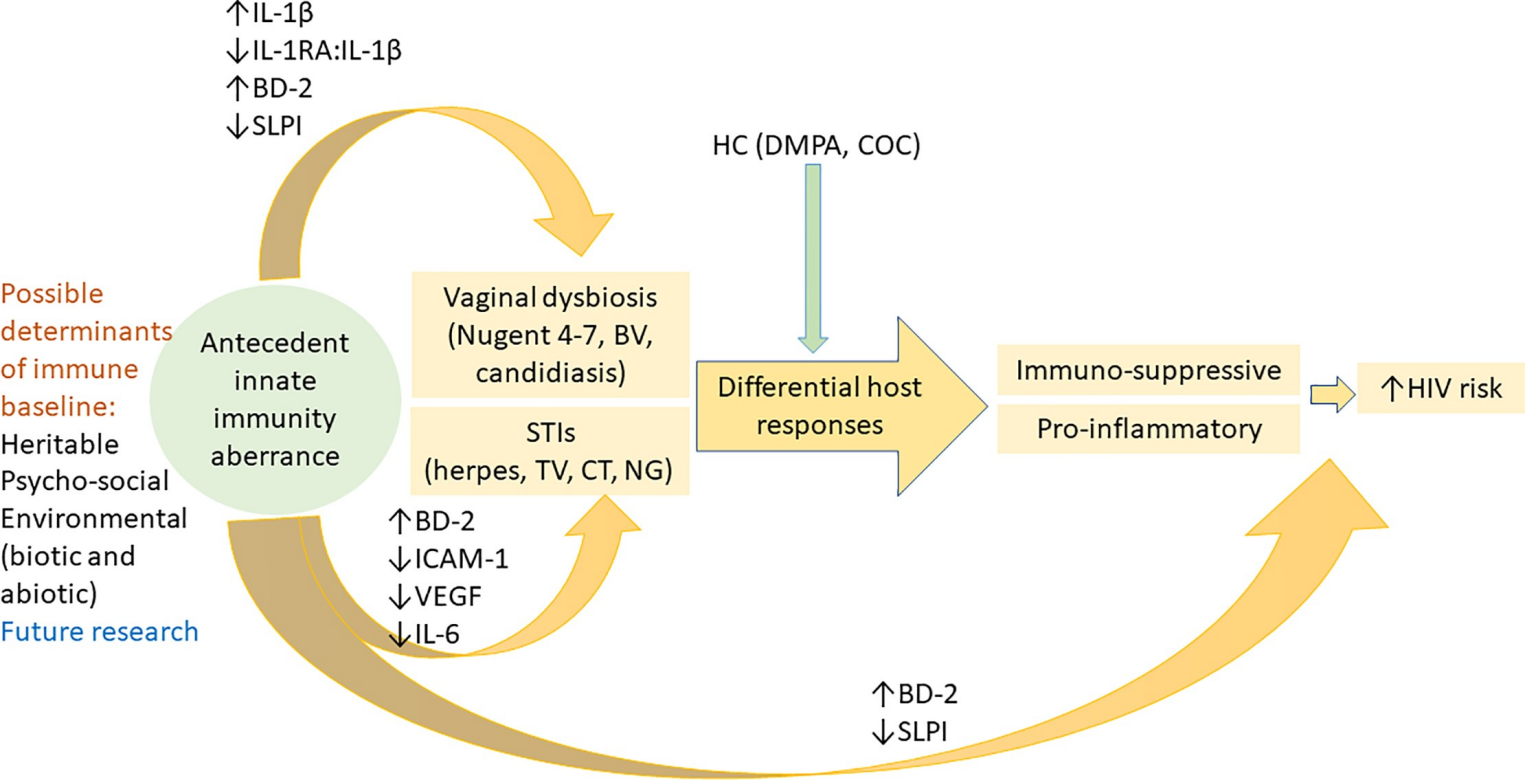
Conceptual summary of the role of prodromal cervical immune aberrance as predictor of dysbiosis and sexually transmitted infections based the evidence from our longitudinal analyses. Immune mediators are featured based on odds ratios for top and bottom quartile concentrations at the visits preceding CVI diagnosis. The independent predictive value of BD-2 and SLPI for HIV seroconversion has been previously established (Morrison et al). Future research must address the determinants of prodromal innate immunity.

Our key findings led us to propose a novel paradigm presented in Fig 4.

We postulate that underexplored genetic and environmental factors including economic and psychosocial stressors [46], behavior [23], systemic factors (e.g., HPV and other viral infections) [28], and/or mucosal microbiome changes [24], not accounted for in this analysis, may shift baseline cervical immunity toward a proinflammatory or immunosuppressive state conducive to cervicovaginal infection. A proinflammatory state, characterized by upregulation of the IL-1 signaling cascade, including IL-1 family members and downstream immune effectors e.g. BD-2, and downregulation of SLPI is particularly predictive of vaginal dysbiosis–manifested by abnormal Nugent scores or candidiasis. On the other hand, increased BD-2 and a shift to an immunosuppressive state characterized mainly by suppressed anti-inflammatory IL-1RA:IL-1β ratio and/or down-regulated ICAM-1, VEGF and IL-6 would predict acquisition of STIs. Once dysbiosis and STI are acquired, cervical responses may be shaped by HC use with a tendency to selective suppression or upregulation of inflammatory responses. For instance, proinflammatory responses to candida prevail in COC users while suppressive responses to bacterial infections are seen in DMPA users. Both shifts in immune responses may facilitate HIV-acquisition. They may also modify the effectiveness of HIV pre-exposure prophylaxis based on the role of well-known mechanisms of vaginal barrier damage and immune activation [47]. Prior analyses within the HC-HIV cohort examined the point prevalence of STIs at the visit prior to that when HIV was detected and associated HIV seroconversion with BV, vaginal yeast infection, trichomoniasis and genital herpes [17, 18]. Similar findings for HSV-2 and trichomoniasis have been reported in other high-risk cohorts [48–50]. In our sample (S1 Fig) HIV-1 risk persisted after trichomoniasis and both bacterial STIs were cleared, raising the possibility that baseline immune imbalance, predisposing to CVIs, may persist after TV, CT and NG clearance and thus may contribute to the risk of HIV in the absence of infection. Microbial factors of persistent immune imbalance may include subtle undiagnosed microbiome transfers between sexual partners. Non-microbial factors resetting cervical immunity after clearance of infection may be of behavioral, environmental or genetic nature. Microbial and non-microbial factors pre-conditioning and resetting cervical immunity require further studies.

In conclusion, our results show that altered genital tract immunity precedes altered microbiota and CVIs. In addition, we show that host responses to dysbiosis and CVIs are modified by HC use, enhancing proinflammatory or immunosuppressive states associated with increased HIV risk. Based on these results, we postulate that an altered cervicovaginal immune status predisposes the cervicovaginal mucosa to a higher risk of clinically significant dysbiosis and possibly prolonged or perturbed clearance of CVI pathogens, which, in turn are known to increase the risk of HIV acquisition. It is important that epidemiological studies and clinical trials, designed to assess HIV risk associated with different types of HC, as well as reproductive hormones in general, vaginal and sexual practices, consider not only the effects of concurrent CVIs but also other undiagnosed or subclinical factors that modify female genital tract immunity prior to infection.

## Supporting information

S1 File Interpretation of supplemental figures and tables. (DOCX)

S1 Table Baseline participant characteristics by prevalent and incident cervicovaginal infections. Baseline is defined either as (A) first of at least 2 consecutive quarterly visits tested for all cervicovaginal infections (CVIs) available for 769 women, or (B) the matched visit preceding the HIV seroconversion by two quarterly visits available for 703 women who were CVI positive at least once during the study.
(XLSX)

S2 Table Distribution of STIs, dysbiosis and co-infection among all HIV-negative visits included in the multivariable analyses. Rates (%) of negative and positive status for each infection (grey-shaded boxes) and by co-infection (clear cells calculated by column) are shown in parentheses.
(DOCX)

S3 Table Mean difference of biomarker concentration between each CVI+ visit and the CVI-free group (n = 134) across all visits controlling for site, age, pregnancy, breastfeeding, overlapping CVIs, unprotected sex and vaginal practice. Yellow shading highlights significant differences between Hormonal contraceptive (HC) use (DMPA and COC) and no HC within each CVI stratum and green shading highlights significant differences between each CVI and CVI-free control within each HC use stratum.
(RTF)

S1 Fig Risk of HIV seroconversion associated with cervicovaginal infections. (DOCX)
