# Supplementary material for: Aberrant cervical innate immunity predicts onset of dysbiosis and sexually transmitted infections in women of reproductive age
Source: PLoS One. 2020 Jan 8;15(1):e0224359. doi: 10.1371/journal.pone.0224359 (PMC6948729; doi:10.1371/journal.pone.0224359)
Supplement: S1 File — (DOCX) [file pone.0224359.s001.docx]

**Supplemental Material**

**S1 File**

**Interpretation of Table S1. Demographic characteristics by cervicovaginal infection (CVI) status**

To compare demographic characteristics between women who acquired CVI during the study to those who never did, and between women who acquired different incident CVIs, we chose two different baseline points – one timed to first of consecutive visits tested for CVI (Table S1 A) and one timed to HIV seroconversion and matched visits (Table S1 B). Table S1 summarizes incident/prevalent CVI definitions (see footnote) and demographics at these two baselines. Only the significant differences are highlighted below:

Country: We found more frequently incident CVIs (20% versus 14%, p=0.031) and, at the matched visit timed to preceding HIV seroconversion visit, more prevalent CVIs (76% versus 24%, p<0.001) and candida (12% versus 5%, p=0.012) among Zimbabwean than Ugandan women.

Age: Prevalent CVIs were more common among older (25+) women (83% versus 71%, p<0.001) and incident CVIs more common among younger (18-24) women (7% versus 4%, p=0.002). At the baseline timed to HIV seroconversion, more of the CVI-positive women were at younger age (18-24 vs 25+) (56% versus 44%, p=0.003) and the HSV-2 seroprevalence was higher among 25+ women (71% versus 48%, p<0.001).

HC use: Among HC users, incident CVIs were more common (22% with COC, and 20% with DMPA versus 12% with non-HC, p<0.013) and prevalent CVIs less common (73% with both COC and DMPA versus 83% with non-HC, p=0.012) compared to non-HC users. HSV-2 seroprevalence was higher among non-HC (66%) compared to DMPA (54%) and COC (56%) users (p=0.016).

Pregnancy and breastfeeding: Intermediary Nugent score (4-6) was most prevalent in the non-pregnant and breastfeeding group (28%), followed by the non-pregnant non-breastfeeding group (24%) and least in the pregnant group (9%) (p=0.044).

Sexual partners and unprotected intercourse: Only 2% of women reported >2 sexual partners at both baselines but only ~1/3 reported sexual abstinence or always protected intercourse. Even though the number of women in the >2 partners’ category was small, trichomoniasis (18% versus 3%, p=0.012), gonorrhea (24% versus 2%, p=0.001) and HSV-2 (82% versus 56%, p=0.046) were more common among them in comparison with women with <2 sexual partners.

Vaginal hygiene practices: Practices of vaginal drying reported at baseline did not influence frequencies of the overall CVI incidence and prevalence during the study. Differences in CVI incidence (p<0.007) but not CVI prevalence (p=0.313) were observed by vaginal cleaning practices, with incident CVI occurring more frequently among women who reported using water only at baseline (23%) versus no cleaning (15%) or other cleaning methods (9-12%). At the matched visit timed to HIV seroconversion, vaginal drying had again no effect for any of the CVIs studied, and vaginal hygiene practices, did not influence frequencies of abnormal Nugent scores, *C. albicans*, and HSV-2. *C. trachomatis* was slightly more frequently detected among women using water or water and soap (3%) compared to no cleaning or other methods (0%). (p=0.025). *T. vaginalis* was more frequent among women using water and soap (7%) or other methods (11%) compared to no cleaning (3%) or water only cleaning (2%) (p<0.015).

**Interpretation of S2 Table**

Rates of all co-infections among all 3087 visits included in the multivariable analyses in this study are shown in Table S2. Co-infections were randomly distributed for each infection with the exception of: 1) gonorrhea which appeared to occur more frequently in co-infection with chlamydiasis compared to overall (14.46% versus 3.08%) and *vice versa* for chlamydiasis, which was more common in coinfection with gonorrhea than overall (12.63% versus 2.69%), and 2) candida which appeared to be less common with BV (Nugent score 7-10) compared to overall (5.86% versus 10.75 %) and *vice versa*, BV – less common with candida compared to overall (17.47% versus 32.04%).

**Interpretation of S3 Table**

Differences biomarker levels between CVI positive and CVI-free visits stratified by HC use including all longitudinal 3274 visits controlling for site, age, pregnancy, breastfeeding, overlapping infections, vaginal practices and unprotected sex confirmed prior findings of HC dependence of host immune responses to prevalent CVIs previously observed with a smaller cross-sectional sample size (1).

**Interpretation of S1 Figure**

The risk of HIV seroconversion by CVI status for the overall HIV-HC study population has been reported elsewhere (2, 3). Comparative OR estimates for HIV risk established by CVIs for the specific set of women examined in this longitudinal study are presented in Supplemental Material and Fig. S1. Generalized linear models were used to estimate the risk (odds ratio (OR) and its 95% confidence interval (CI) and p value) of HIV seroconversion (visit T-0) for women who were positive for a particular cervicovaginal infection (CVI) at one or two prior consecutive quarterly visits preceding HIV seroconversion (T-2 and/or T-1) compared to women who were CVI-free. CVI-free is defined as Nugent <4 and no positive result for any of the pathogens listed in each plot at both T-2 and T-1 visits. P values are based on risk order by (T-2, T-1) : 0=(0,0); 1= (1,0); 2= (0,1); 3= (1,1) adjusted by site, age, use of hormonal contraception, pregnancy, breastfeeding, overlapping CVIs, number of sexual partners, unprotected sex acts, and vaginal hygiene practices) at the visit closest to HIV seroconversion (T-1 visit). Of note, novel finding, HIV-1 risk persisted after trichomoniasis and both bacterial STIs were cleared, supporting the hypothesis that baseline immune imbalance.

The sample size for this analyses included a total 825 women of those 200 who HIV seroconverted with:

• CVI[+] at T-2 and CVI [-] at T-1 visit: BV (abnormal Nugent n=86); *T. vaginalis* (TV) (n=15); Candida (n=46); Chlamydia (n=10); *N. gonorrhoeae* (n=11); HSV (n=0).

• CVI[-] at T-2 and CVI [+] at T-1 visit: BV (abnormal Nugent n=110); TV (n=25); Candida (n=69); Chlamydia (n=21); *N. gonorrhoeae* (n=27); HSV (n=11).

• CVI[+] at T-2 and CVI [+] at T-1 visit: BV (abnormal Nugent n=235); TV (n=4); Candida (n=19); Chlamydia (n=5); *N. gonorrhoeae* (n=8); HSV (n=239).

**References**

1. Fichorova RN, Chen PL, Morrison CS, Doncel GF, Mendonca K, Kwok C, et al. The Contribution of Cervicovaginal Infections to the Immunomodulatory Effects of Hormonal Contraception. MBio. 2015;6(5):e00221-15.

2. Van Der Pol B, Kwok C, Pierre-Louis B, Rinaldi A, Salata RA, Chen PL, et al. Trichomonas vaginalis infection and human immunodeficiency virus acquisition in African women. The Journal of infectious diseases. 2008;197(4):548-54.

3. van de Wijgert JH, Morrison CS, Cornelisse PG, Munjoma M, Moncada J, Awio P, et al. Bacterial vaginosis and vaginal yeast, but not vaginal cleansing, increase HIV-1 acquisition in African women. J Acquir Immune Defic Syndr. 2008;48(2):203-10.
