# Supplementary material for: Aberrant cervical innate immunity predicts onset of dysbiosis and sexually transmitted infections in women of reproductive age
Source: PLoS One. 2020 Jan 8;15(1):e0224359. doi: 10.1371/journal.pone.0224359 (PMC6948729; doi:10.1371/journal.pone.0224359)
Supplement: S2 Table — Rates (%) of negative and positive status for each infection (grey-shaded boxes) and by co-infection (clear cells calculated by column) are shown in parentheses. (DOCX) [file pone.0224359.s003.docx]

**Table S2.** Distribution of STIs, dysbiosis and co-infection among all HIV-negative visits included in the multivariable analyses (n=3087). Rates (%) of negative and positive status for each infection (grey- shaded boxes) and by co-infection (clear cells calculated by column) are shown in paretheses.

|  | | ***T. vaginalis* infection** | | **Chlamydia** | | **Candidiasis** | | **Gonorrhea** | | **Nugent Score** | | | **HSV-2** | |
| --- | --- | --- | --- | --- | --- | --- | --- | --- | --- | --- | --- | --- | --- | --- |
| **Parameter** |  | - | + | - | + | - | + | - | + | 0-3 | 4-6 | 7-10 | - | + |
| ***T. vaginalis* infection** | - | 2991 (96.89) |  | 2912 (96.94) | 79 (95.18) | 2668 (96.84) | 323 (97.29) | 2904 (97.06) | 87 (91.58) | 1424 (98.41) | 619 (95.08) | 948 (95.85) | 1277 (97.56) | 1714 (96.40) |
|  | + |  | 96 (3.11) | 92 (3.06) | 4 (4.82) | 87 (3.16) | 9 (2.71) | 88 (2.94) | 8 (8.42) | 23 (1.59) | 32 (4.92) | 41 (4.15) | 32 (2.44) | 64 (3.60) |
| **Chlamydia** | - | 2912 (97.36) | 92 (95.83) | 3004 (97.31) |  | 2676 (97.13) | 328 (98.80) | 2921 (97.63) | 83 (87.37) | 1419 (98.06) | 637 (97.85) | 948 (95.85) | 1282 (97.94) | 1722 (96.85) |
|  | + | 79 (2.64) | 4 (4.17) |  | 83 (2.69) | 79 (2.87) | 4 (1.20) | 71 (2.37) | 12 (12.63) | 28 (1.94) | 14 (2.15) | 41 (4.15) | 27 (2.06) | 56 (3.15) |
| **Candidiasis** | - | 2668 (89.20) | 87 (90.63) | 2676 (89.08) | 79 (95.18) | 2755 (89.25) |  | 2670 (89.24) | 85 (89.47) | 1261 (87.15) | 563 (86.48) | 931 (94.14) | 1167 (89.15) | 1588 (89.31) |
|  | + | 323 (10.80) | 9 (9.38) | 328 (10.92) | 4 (4.82) |  | 332 (10.75) | 322 (10.76) | 10 (10.53) | 186 (12.85) | 88 (13.52) | 58 (5.86) | 142 (10.85) | 190 (10.69) |
| **Gonorrhea** | - | 2904 (97.09) | 88 (91.67) | 2921 (97.24) | 71 (85.54) | 2670 (96.91) | 322 (96.99) | 2992 (96.92) |  | 1418 (98.00) | 631 (96.93) | 943 (95.35) | 1280 (97.78) | 1712 (96.29) |
|  | + | 87 (2.91) | 8 (8.33) | 83 (2.76) | 12 (14.46) | 85 (3.09) | 10 (3.01) |  | 95 (3.08) | 29 (2.00) | 20 (3.07) | 46 (4.65) | 29 (2.22) | 66 (3.71) |
| **Nugent Score** | 0-3 | 1424 (47.61) | 23 (23.96) | 1419 (47.24) | 28 (33.73) | 1261 (45.77) | 186 (56.02) | 1418 (47.39) | 29 (30.53) | 1447 (46.87) |  |  | 682 (52.10) | 765 (43.03) |
|  | 4-6 | 619 (20.70) | 32 (33.33) | 637 (21.21) | 14 (16.87) | 563 (20.44) | 88 (26.51) | 631 (21.09) | 20 (21.05) |  | 651 (21.09) |  | 252 (19.25) | 399 (22.44) |
|  | 7-10 | 948 (31.70) | 41 (42.71) | 948 (31.56) | 41 (49.40) | 931 (33.79) | 58 (17.47) | 943 (31.52) | 46 (48.42) |  |  | 989 (32.04) | 375 (28.65) | 614 (34.53) |
| **HSV-2** | - | 1277 (42.69) | 32 (33.33) | 1282 (42.68) | 27 (32.53) | 1167 (42.36) | 142 (42.77) | 1280 (42.78) | 29 (30.53) | 682 (47.13) | 252 (38.71) | 375 (37.92) | 1309 (42.40) |  |
|  | + | 1714 (57.31) | 64 (66.67) | 1722 (57.32) | 56 (67.47) | 1588 (57.64) | 190 (57.23) | 1712 (57.22) | 66 (69.47) | 765 (52.87) | 399 (61.29) | 614 (62.08) |  | 1778 (57.60) |
