# Supplementary material for: Aberrant cervical innate immunity predicts onset of dysbiosis and sexually transmitted infections in women of reproductive age
Source: PLoS One. 2020 Jan 8;15(1):e0224359. doi: 10.1371/journal.pone.0224359 (PMC6948729; doi:10.1371/journal.pone.0224359)
Supplement: S3 Table — Yellow shading highlights significant differences between Hormonal contraceptive (HC) use (DMPA and COC) and no HC within each CVI stratum and green shading highlights significant differences between each CVI and CVI-free control within each HC use stratum. (RTF) [file pone.0224359.s004.rtf]

CVI	IL-1â	IL-6	IL-8	VEGF	MIP-3á	
CVI-1						
no HC (pg/mg mean, SE)	3.31 (0.56)	16.92 (2.59)	554.89 (131.09)	1094.93 (128.05)	284.60 (40.81)	
DMPA (difference, p)	0.38 (0.7004)	-1.97 (0.6123)	-99.16 (0.6312)	176.66 (0.4183)	-21.26 (0.7301)	
COC (difference, p)	0.45 (0.6574)	-0.45 (0.9104)	90.16 (0.7028)	185.41 (0.4040)	57.52 (0.4090)	
Nugent 7-101						
no HC (pg/mg mean, SE)	3.85 (0.24)	16.21 (0.81)	619.76 (45.92)	1153.68 (44.63)	328.26 (17.93)	
DMPA (difference, p)	0.51 (0.1911)	2.62 (0.0464)	138.32 (0.0661)	104.82 (0.1299)	-22.70 (0.3785)	
COC (difference, p)	1.23 (0.0038)	5.34 (0.0002)	311.11 (0.0002)	125.65 (0.0721)	86.32 (0.0050)	
Nugent 7-10 vs. CVI-						
no HC (difference, p)	-0.15 (0.8472)	-2.98 (0.3073)	-33.23 (0.8342)	68.61 (0.6265)	28.84 (0.6079)	
DMPA (difference, p)	-0.02 (0.9851)	0.14 (0.9626)	248.88 (0.0907)	-12.71 (0.9320)	-5.05 (0.9221)	
COC (difference, p)	0.63 (0.4942)	3.57 (0.2541)	273.69 (0.1346)	5.46 (0.9719)	71.07 (0.2847)	
Nugent 4-61						
no HC (pg/mg mean, SE)	4.62 (0.32)	17.60 (1.08)	660.87 (58.43)	1230.19 (57.48)	296.82 (20.23)	
DMPA (difference, p)	0.60 (0.2334)	1.39 (0.4041)	225.16 (0.0237)	-33.82 (0.6799)	12.07 (0.6955)	
COC (difference, p)	0.34 (0.4924)	4.02 (0.0250)	244.61 (0.0145)	50.33 (0.5534)	99.99 (0.0049)	
Nugent 4-6 vs. CVI-						
no HC (difference, p)	0.66 (0.4058)	-0.47 (0.8758)	66.12 (0.6802)	213.84 (0.1234)	27.62 (0.6047)	
DMPA (difference, p)	0.49 (0.5532)	-0.79 (0.7887)	324.46 (0.0395)	-105.96 (0.4448)	-4.17 (0.9379)	
COC (difference, p)	0.31 (0.7020)	2.69 (0.3950)	227.26 (0.1980)	25.59 (0.8612)	50.55 (0.4473)	

CVI	IL-1â	IL-6	IL-8	VEGF	MIP-3á	
T. vaginalis1						
no HC (pg/mg mean, SE)	4.11 (0.63)	N/A	843.80 (146.57)	1204.52 (133.55)	338.96 (40.78)	
DMPA (difference, p)	1.23 (0.3714)	N/A	280.82 (0.3858)	78.90 (0.7571)	-8.63 (0.9097)	
COC (difference, p)	0.32 (0.7998)	N/A	33.11 (0.9107)	-21.56 (0.9308)	175.52 (0.0847)	
T. vaginalis vs. CVI-						
no HC (difference, p)	1.01 (0.6874)	-4.94 (0.5866)	47.28 (0.9366)	-341.11 (0.4947)	-161.25 (0.2988)	
DMPA (difference, p)	2.09 (0.5124)	2.26 (0.8370)	615.46 (0.3543)	-356.04 (0.5644)	-215.58 (0.1677)	
COC (difference, p)	0.70 (0.8025)	-1.29 (0.8961)	221.93 (0.7355)	-475.07 (0.4127)	-205.48 (0.3174)	
Candida1						
no HC (pg/mg mean, SE)	5.54 (0.52)	20.60 (1.67)	901.97 (103.60)	1332.14 (84.49)	356.89 (31.96)	
DMPA (difference, p)	0.92 (0.3036)	1.91 (0.4912)	119.77 (0.4931)	-36.43 (0.7754)	32.89 (0.5396)	
COC (difference, p)	1.43 (0.1169)	9.16 (0.0043)	660.26 (0.0016)	217.37 (0.1156)	291.10 (<.0001)	
Candida vs. CVI-						
no HC (difference, p)	2.10 (0.0295)	2.68 (0.4538)	385.80 (0.0660)	398.98 (0.0154)	69.69 (0.2712)	
DMPA (difference, p)	2.24 (0.0238)	4.78 (0.1622)	558.87 (0.0025)	114.39 (0.4740)	92.05 (0.1468)	
COC (difference, p)	2.75 (0.0075)	11.77 (0.0039)	974.12 (0.0002)	415.08 (0.0226)	330.97 (0.0005)	
Chlamydia1						
no HC (pg/mg mean, SE)	3.95 (0.75)	22.40 (4.00)	798.16 (187.12)	1194.00 (181.44)	348.56 (75.92)	
DMPA (difference, p)	-0.06 (0.9582)	-1.33 (0.8346)	-146.13 (0.6113)	-262.18 (0.2935)	-85.54 (0.4541)	
COC (difference, p)	0.72 (0.5700)	-6.85 (0.1760)	51.11 (0.8643)	-162.63 (0.5121)	-22.25 (0.8530)	

CVI	IL-1â	IL-6	IL-8	VEGF	MIP-3á	
Chlamydia vs. CVI-						
no HC (difference, p)	1.91 (0.3847)	22.27 (0.0403)	585.62 (0.2697)	552.99 (0.2302)	243.07 (0.1772)	
DMPA (difference, p)	1.90 (0.3752)	26.94 (0.0113)	520.37 (0.2237)	123.89 (0.7707)	221.24 (0.1527)	
COC (difference, p)	2.57 (0.2181)	16.74 (0.0267)	625.42 (0.1896)	278.79 (0.4853)	225.33 (0.1643)	
Gonorrhea1						
no HC (pg/mg mean, SE)	3.93 (0.82)	17.57 (3.06)	720.35 (141.15)	1146.71 (129.22)	409.64 (68.17)	
DMPA (difference, p)	-0.31 (0.8155)	-0.65 (0.8947)	-125.17 (0.5473)	-173.54 (0.3265)	-135.16 (0.1299)	
COC (difference, p)	1.13 (0.5266)	-0.60 (0.9141)	258.59 (0.3746)	-22.99 (0.9149)	-56.55 (0.6203)	
Gonorrhea vs. CVI-						
no HC (difference, p)	-1.01 (0.5804)	-12.94 (0.0841)	28.33 (0.9413)	-128.20 (0.7031)	0.38 (0.9978)	
DMPA (difference, p)	-0.42 (0.8201)	-10.64 (0.0838)	-80.05 (0.7804)	-367.65 (0.2269)	-88.22 (0.3782)	
COC (difference, p)	0.51 (0.8300)	-12.47 (0.0897)	-2.69 (0.9953)	-273.37 (0.4619)	-119.23 (0.4026)	
HSV21						
no HC (pg/mg mean, SE)	4.32 (0.21)	16.60 (0.67)	660.67 (39.78)	1212.50 (37.19)	334.75 (15.39)	
DMPA (difference, p)	0.35 (0.2679)	1.27 (0.2075)	106.16 (0.0823)	-36.65 (0.4818)	-17.46 (0.4183)	
COC (difference, p)	0.78 (0.0203)	4.09 (0.0002)	262.10 (<.0001)	53.70 (0.3268)	95.78 (0.0002)	
HSV2 vs. CVI-						
no HC (difference, p)	0.72 (0.3314)	-1.46 (0.5890)	78.09 (0.5969)	166.56 (0.2033)	60.93 (0.2519)	
DMPA (difference, p)	0.70 (0.3336)	-0.59 (0.8179)	275.60 (0.0417)	-106.47 (0.4110)	22.36 (0.6477)	
COC (difference, p)	1.23 (0.1167)	3.42 (0.2216)	302.73 (0.0708)	6.14 (0.9650)	113.50 (0.0722)	

CVI	RANTES	BD2	SLPI	IL-1RA	IL-1RA:IL1â	ICAM-1	
CVI-1							
no HC (pg/mg mean, SE)	19.20 (2.68)	1151.86 (344.72)	83649.4 (18639.5)	805.54 (76.16)	226.79 (28.02)	365.88 (54.52)	
DMPA (difference, p)	15.29 (0.0261)	-447.52 (0.4363)	2956.82 (0.9323)	103.78 (0.4241)	1.57 (0.9721)	137.05 (0.1645)	
COC (difference, p)	2.26 (0.6480)	-350.66 (0.5744)	53276.6 (0.2548)	59.38 (0.6456)	14.87 (0.7542)	55.29 (0.5544)	
Nugent 7-101							
no HC (pg/mg mean, SE)	33.49 (1.98)	1766.02 (192.28)	111038 (9468.63)	848.30 (26.73)	213.72 (10.08)	419.16 (22.99)	
DMPA (difference, p)	18.92 (<.0001)	-107.37 (0.7189)	-25156 (0.0516)	-38.85 (0.3124)	-25.83 (0.0634)	53.53 (0.1330)	
COC (difference, p)	2.07 (0.5137)	-507.26 (0.0478)	53216.0 (0.0035)	-15.27 (0.6963)	-54.94 (<.0001)	-6.58 (0.8449)	
Nugent 7-10 vs. CVI-							
no HC (difference, p)	7.44 (0.1691)	-395.94 (0.6015)	22641.4 (0.4184)	90.62 (0.2630)	27.38 (0.3758)	19.87 (0.7860)	
DMPA (difference, p)	-2.67 (0.7968)	931.62 (0.0145)	-51010 (0.0728)	-8.34 (0.9112)	-8.35 (0.7496)	-66.61 (0.4065)	
COC (difference, p)	3.94 (0.4981)	438.05 (0.2187)	-30856 (0.5235)	55.57 (0.4705)	-44.01 (0.0758)	-38.75 (0.5913)	
Nugent 4-61							
no HC (pg/mg mean, SE)	40.15 (3.17)	1914.50 (268.38)	81721.2 (9430.02)	793.40 (31.70)	180.63 (9.56)	452.17 (28.97)	
DMPA (difference, p)	8.30 (0.1300)	542.77 (0.2883)	15186.3 (0.3486)	-14.32 (0.7526)	-18.34 (0.1625)	2.83 (0.9465)	
COC (difference, p)	-3.73 (0.4097)	-565.98 (0.1155)	104153 (<.0001)	37.11 (0.4318)	-8.10 (0.5528)	3.51 (0.9331)	
Nugent 4-6 vs. CVI-							
no HC (difference, p)	11.16 (0.0894)	362.87 (0.6161)	-12283 (0.6635)	60.76 (0.4493)	-8.61 (0.7498)	22.78 (0.7584)	
DMPA (difference, p)	-6.94 (0.4865)	1674.81 (0.0014)	-70503 (0.0656)	-63.90 (0.4100)	-28.70 (0.2025)	-145.89 (0.0590)	
COC (difference, p)	4.15 (0.4913)	511.08 (0.2022)	-61269 (0.3464)	50.36 (0.5304)	-22.52 (0.3621)	-48.64 (0.5094)	

CVI	RANTES	BD2	SLPI	IL-1RA	IL-1RA:IL1â	ICAM-1	
T. vaginalis1							
no HC (pg/mg mean, SE)	47.30 (7.95)	8200.92 (2111.32)	104039 (24755.9)	748.98 (66.67)	164.84 (17.06)	387.42 (56.65)	
DMPA (difference, p)	16.09 (0.4083)	-5704.0 (0.0260)	-34989 (0.4095)	-30.92 (0.7870)	-0.35 (0.9910)	-57.75 (0.5346)	
COC (difference, p)	-13.08 (0.3335)	-5029.8 (0.0905)	226288 (0.0323)	151.53 (0.2724)	54.09 (0.1277)	-95.78 (0.2982)	
T. vaginalis vs. CVI-							
no HC (difference, p)	9.65 (0.6118)	10226.3 (0.1375)	-82125 (0.3855)	-222.12 (0.4306)	-127.71 (0.1758)	-56.61 (0.8028)	
DMPA (difference, p)	-3.80 (0.9051)	4846.44 (0.0843)	-203875 (0.0433)	-405.82 (0.1878)	-161.53 (0.0533)	-235.28 (0.3016)	
COC (difference, p)	-1.21 (0.9410)	7663.24 (0.0621)	-226442 (0.2749)	-221.44 (0.5097)	-138.25 (0.1692)	-161.62 (0.4325)	
Candida1							
no HC (pg/mg mean, SE)	30.65 (2.89)	3626.99 (655.00)	124607 (17629.0)	865.57 (46.95)	167.15 (12.60)	370.26 (35.82)	
DMPA (difference, p)	34.87 (<.0001)	-149.42 (0.8981)	-9416.7 (0.7425)	19.72 (0.7849)	-12.77 (0.4972)	142.83 (0.0266)	
COC (difference, p)	11.25 (0.0480)	-492.17 (0.6461)	220962 (<.0001)	60.02 (0.4089)	-30.78 (0.0710)	169.59 (0.0083)	
Candida vs. CVI-							
no HC (difference, p)	3.92 (0.4850)	2356.43 (0.0389)	20568.3 (0.5444)	174.77 (0.0534)	-31.57 (0.2963)	11.97 (0.8732)	
DMPA (difference, p)	-0.96 (0.9395)	3002.28 (<.0001)	-42686 (0.2499)	39.22 (0.6649)	-58.04 (0.0360)	-72.34 (0.4324)	
COC (difference, p)	3.60 (0.6114)	2541.50 (0.0003)	93562.6 (0.2445)	147.45 (0.1147)	-88.14 (0.0019)	48.27 (0.5960)	
Chlamydia1							
no HC (pg/mg mean, SE)	47.51 (10.28)	1639.72 (455.93)	188573 (57732.5)	937.10 (138.48)	270.74 (43.72)	451.41 (88.59)	
DMPA (difference, p)	28.16 (0.2729)	-276.71 (0.7359)	-95993 (0.2516)	-357.65 (0.0302)	-93.73 (0.0920)	2.31 (0.9866)	
COC (difference, p)	-8.30 (0.5932)	1389.11 (0.2564)	-129181 (0.0398)	-254.76 (0.1447)	-123.56 (0.0095)	-143.49 (0.2073)	

CVI	RANTES	BD2	SLPI	IL-1RA	IL-1RA:IL1â	ICAM-1	
Chlamydia vs. CVI-							
no HC (difference, p)	39.86 (0.0378)	426.75 (0.8359)	401612 (0.0179)	473.83 (0.1635)	48.09 (0.6437)	279.92 (0.1896)	
DMPA (difference, p)	65.38 (0.0841)	479.76 (0.6288)	198765 (0.0901)	20.75 (0.9329)	-36.76 (0.6432)	247.16 (0.2939)	
COC (difference, p)	27.46 (0.0698)	1521.43 (0.3322)	149023 (0.1659)	178.02 (0.4448)	-71.31 (0.2696)	146.31 (0.3755)	
Gonorrhea1							
no HC (pg/mg mean, SE)	32.50 (5.13)	2848.24 (714.67)	118651 (30365.9)	812.41 (76.20)	202.50 (32.41)	311.99 (50.70)	
DMPA (difference, p)	27.08 (0.0357)	-1223.0 (0.2182)	-56255 (0.1683)	-152.63 (0.1260)	-22.09 (0.6573)	60.06 (0.4675)	
COC (difference, p)	5.19 (0.6194)	-63.91 (0.9660)	-8209.7 (0.8925)	-53.07 (0.6636)	-80.07 (0.0787)	51.82 (0.5758)	
Gonorrhea vs. CVI-							
no HC (difference, p)	1.30 (0.8985)	2777.04 (0.1732)	-97429 (0.1516)	-4.43 (0.9828)	27.27 (0.7529)	-177.88 (0.2116)	
DMPA (difference, p)	-7.46 (0.7082)	1709.69 (0.1166)	-127939 (0.0133)	-190.12 (0.2690)	-14.63 (0.8408)	-310.57 (0.0361)	
COC (difference, p)	-1.19 (0.9224)	3238.54 (0.0917)	-187687 (0.0516)	-31.16 (0.8829)	-89.94 (0.1989)	-214.66 (0.1743)	
HSV21							
no HC (pg/mg mean, SE)	34.47 (1.67)	1996.55 (184.80)	109282 (8181.88)	839.97 (20.88)	193.15 (6.84)	435.46 (19.93)	
DMPA (difference, p)	22.98 (<.0001)	-134.74 (0.6193)	-6936.2 (0.5569)	-53.02 (0.0650)	-18.26 (0.0497)	38.08 (0.1920)	
COC (difference, p)	1.87 (0.4683)	-538.25 (0.0239)	75211.4 (<.0001)	1.67 (0.9561)	-29.98 (0.0009)	-1.62 (0.9544)	
HSV2 vs. CVI-							
no HC (difference, p)	10.75 (0.0353)	476.33 (0.4652)	24335.7 (0.3888)	67.94 (0.3675)	-14.90 (0.5899)	92.35 (0.1819)	
DMPA (difference, p)	6.40 (0.5254)	1264.35 (0.0005)	-41430 (0.1986)	-91.54 (0.1869)	-50.59 (0.0328)	-32.76 (0.6679)	
COC (difference, p)	7.45 (0.1720)	772.85 (0.0255)	-12085 (0.8203)	17.70 (0.8103)	-71.62 (0.0037)	2.99 (0.9665)	
