# Supplementary material for: Aberrant cervical innate immunity predicts onset of dysbiosis and sexually transmitted infections in women of reproductive age
Source: PLoS One. 2020 Jan 8;15(1):e0224359. doi: 10.1371/journal.pone.0224359 (PMC6948729; doi:10.1371/journal.pone.0224359)
Supplement: S1 Fig — (DOCX) [file pone.0224359.s005.docx]

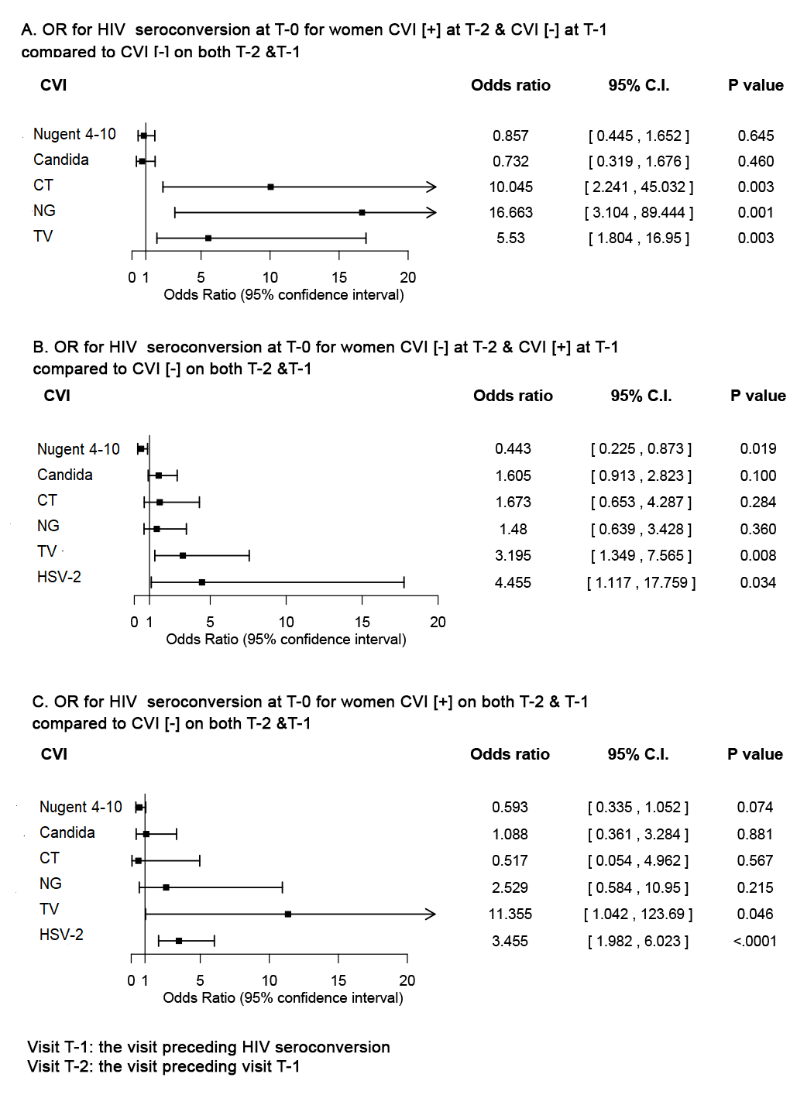


**S1 Fig: Risk of HIV seroconversion associated with cervicovaginal infections (CVI).** Generalized linear models were used to estimate the risk (odds ratio (OR) and its 95% confidence interval (CI) and p value) of HIV seroconversion (visit T-0) for women who were positive for a particular cervicovaginal infection (CVI) at one or two prior consecutive quarterly visits preceding HIV seroconversion (T-2 and/or T-1) compared to women who were CVI-free. CVI-free is defined as Nugent <4 and no positive result for any of the pathogens listed in each plot at both T-2 and T-1 visits. P values are based on risk order by (T-2, T-1) : 0=(0,0); 1= (1,0); 2= (0,1); 3= (1,1) adjusted by site, age, use of hormonal contraception, pregnancy, breastfeeding, overlapping CVIs, number of sexual partners, unprotected sex acts, and vaginal hygiene practices) at the visit closest to HIV seroconversion (T-1 visit). CT- *C. trachomatis*, NG – *N. gonorrhoeae*, TV – *T. vaginalis*. Number of cases with CVIs preceding HIV seroconversion: A) CVI+ at T-2 and CVI- at T-1: Nugent 4-10=44; Candida=9; CT=6, NG=9, TV=9; B) CVI- at T-2; and CVI+ at T-1: Nugent 4-10=49, Candida=27, CT=9, TV=14, HSV-2=5; C) CVI+ at both T-2 and T-1: Nugent 4-10=39, Candida=5, CT=1, NG=4, TV=3, HSV-2=124.
